# IL-10 signaling in CD4^+ ^T cells is critical for the pathogenesis of collagen-induced arthritis

**DOI:** 10.1186/ar3545

**Published:** 2011-12-22

**Authors:** Jian Tao, Masahito Kamanaka, Jianlei Hao, Zhifang Hao, Xi Jiang, Joe E Craft, Richard A Flavell, Zhenzhou Wu, Zhangyong Hong, Liqing Zhao, Zhinan Yin

**Affiliations:** 1 Section of Rheumatology, Department of Internal Medicine, Yale School of Medicine, 333 Cedar Street, New Haven, CT 06520-8031, USA; 2 Department of Immunobiology, Yale School of Medicine, 333 Cedar Street, New Haven, CT 06520-8031, USA; 3 College of Life Sciences, Nankai University, 94 Weijin Road, Tianjin, 300071, China; 4 Department of Surgery, University of Connecticut Health Center, 263 Farmington Avenue, Farmington, CT 06030, USA; 5 Department of Reconstructive Sciences, University of Connecticut Health Center, 263 Farmington Avenue, Farmington, CT 06030, USA

## Abstract

**Introduction:**

IL-10 is a very important anti-inflammatory cytokine. However, the role of this cytokine in T cells in the pathogenesis of collagen-induced arthritis is unclear. The purpose of this study was to define the role of IL-10 signaling in T cells in the pathogenesis of collagen-induced arthritis.

**Methods:**

IL-10 receptor dominant-negative transgenic (Tg) and control mice were immunized with bovine type II collagen to induce arthritis. The severity of arthritis was monitored and examined histologically. T-cell activation and cytokine production were analyzed using flow cytometry. T-cell proliferation was examined by [^3^H]thymidine incorporation. Antigen-specific antibodies in serum were measured by ELISA. Foxp3 expression in CD4^+ ^regulatory T cells (Tregs) was determined by intracellular staining or Foxp3-RFP reporter mice. The suppressive function of Foxp3^+^CD4^+ ^Tregs was determined *in vitro *by performing a T-cell proliferation assay. The level of IL-17 mRNA in joints was measured by real-time PCR. A two-tailed nonparametric paired test (Wilcoxon signed-rank test) was used to calculate the arthritis and histological scores. Student's paired or unpaired *t*-test was used for all other statistical analyses (InStat version 2.03 software; GraphPad Software, San Diego, CA, USA).

**Results:**

Blocking IL-10 signaling in T cells rendered mice, especially female mice, highly susceptible to collagen-induced arthritis. T-cell activation and proliferation were enhanced and produced more IFN-γ. The suppressive function of CD4^+^Foxp3^+ ^regulatory T cells was significantly impaired in Tg mice because of the reduced ability of Tregs from Tg mice to maintain their levels of Foxp3. This was further confirmed by transferring Foxp3-RFP cells from Tg or wild-type (Wt) mice into a congenic Wt host. The higher level of IL-17 mRNA was detected in inflammatory joints of Tg mice, probably due to the recruitment of IL-17^+^γδ T cells into the arthritic joints.

**Conclusion:**

IL-10 signaling in T cells is critical for dampening the pathogenesis of collagen-induced arthritis by maintaining the function of Tregs and the recruitment of IL-17^+^γδ T cells.

## Introduction

Rheumatoid arthritis (RA) is an autoimmune disease characterized by chronic inflammation of the joint capsule and synovial membrane which results in cartilage injury, bone erosion and eventually joint destruction and deformity [1]. Collagen-induced arthritis (CIA) is a well-established animal model that has been studied extensively because of its similarities to human RA [2]. Although the etiology of RA remains unknown, it has been reported that a functional imbalance between proinflammatory cytokines and regulatory T cells (Tregs) is a key mechanism that underlies joint inflammation and disease progression in CIA as well as RA [3].

IL-10 is a pleiotropic cytokine with important immune-regulatory functions [4]. It has been implicated to have a potent anti-inflammatory role in several autoimmune disease models, including CIA [5]. IL-10 suppresses the expression of inflammatory cytokines such as TNF-α, IL-6 and IL-1 by activated macrophages [6]. IL-10 also affects T-cell proliferation and cytokine production [7,8]. Indeed, IL-10 affects many of the cell types in the immune system; however, the precise role of IL-10 signaling in CD4^+ ^T cells in the pathogenesis of CIA has not been addressed.

It has been demonstrated in both animal model and human studies that naturally occurring CD4^+^CD25^+^Foxp3^+ ^Tregs play a critical role in the prevention of autoimmunity and inflammatory arthritis [9,10]. Depletion of CD4^+^CD25^+ ^T cells aggravated CIA [11,12], whereas transferring CD4^+^CD25^+ ^cells to a disease-bearing animal ameliorated arthritis [12]. Although it has been well-documented that IL-10 can induce differentiation of naïve CD4^+ ^T cells into CD4^+^IL-10^+ ^Tr1 cells [13,14], it is unclear whether IL-10 signaling in T cells affects the development or function of these Tregs.

Recently, a new subset of IL-17-producing CD4^+ ^T cells, also called Th17 cells [15,16], has been implicated as an important mediator in tissue inflammation [17]. IL-17-deficient mice showed markedly suppressed CIA [18]. Resistance to CIA in p19^-/- ^mice correlated with an absence of IL-17-producing CD4^+ ^T cells, suggesting that the IL-23-IL-17 axis rather than the IL-12-IFN-γ axis is essential in promoting the development of CIA [19]. It has been reported that IL-10 suppresses Th17 cytokine secretion by macrophages and T cells in *in vitro *culture [20]. However, the role of IL-10 signaling in T cells in the differentiation of Th17 cells and how this regulation affects the pathogenesis of CIA is less clear.

In this study, in which we studied previously described IL-10 receptor dominant-negative transgenic (Tg) mice [21], we showed that when T cells fail to respond to IL-10, mice develop more severe arthritis and T cells are more activated and proliferate more against type II collagen (CII) antigen. Moreover, the suppressive function of Tregs in these Tg mice was significantly impaired as a result of attenuated expression of Foxp3. IL-10 signaling in CD4^+ ^T cells might affect the cytokine profiles of CD4^+ ^T cells as well as γδ T cells. These results indicate that IL-10 signaling in T cells is the key to the pathogenesis of CIA.

## Materials and methods

### Mice

DBA/1 and C57BL/6 mice (Thy1.2 or Thy1.1 background) were purchased from The Jackson Laboratory (Bar Harbor, ME, USA) and maintained under specific pathogen-free conditions at the Yale University School of Medicine. DBA/1 background IL-10 receptor dominant-negative Tg mice were obtained by backcrossing C57BL/6 Tg mice [21] with wild-type (Wt) DBA/1 mice for nine generations. The H-2^q/q ^and Tg genotypes were screened by PCR using tail DNA. The IL-10 GFP knockin mouse, designated IL-10-internal ribosomal entry site (IRES)-GFP-enhanced reporter (Tiger), and the Foxp3 red fluorescent protein (RFP) knockin mouse, designated Foxp3-IRES-mRFP (FIR) mice, were described previously [22,23]. All the animal procedures were performed with the approval of the Institutional Animal Care and Use Committee of the Yale University School of Medicine.

### Reagents

Bovine CII for immunization, T-cell proliferation and ELISA, as well as Complete Freund's Adjuvant (CFA) and Incomplete Freund's Adjuvant (IFA), were all purchased from Chondrex (Redmond, WA, USA). BD GolgiPlug and antibodies for flow cytometry were purchased from BD (San Diego, CA, USA). A Foxp3 intracellular staining kit was purchased from eBioscience (San Diego, CA, USA). Cytokines and antibodies for T-cell differentiation *in vitro *were obtained from R&D Systems (Minneapolis, MN, USA). Phorbol 12-myristate 13-acetate (PMA) and ionomycin were purchased from Sigma-Aldrich (St Louis, MO, USA).

### Collagen-induced arthritis model

The CIA model was employed as described previously [24]. Briefly, 100 μg of bovine CII dissolved in 0.1 M acetic acid was emulsified with 100 μl of CFA containing 2 mg/ml inactivated *Mycobacterium tuberculosis*. Mice were sensitized by intradermal injection at the base of the tail and boosted at day 21 with the same dose of CII and IFA intradermally. Mice were scored blindly for disease severity every other day after the boost using a grading scale from 0 to 3 that was described previously [24]. Each paw was graded with a maximum score of 12 per mouse.

### Cell proliferation

Splenocytes from CIA mice were cultured with different concentrations of CII for 3 days. After pulsing with 1 μCi of [^3^H]thymidine per well for the last 18 hours, proliferation was measured as radioactivity incorporation (counts per minute (cpm)).

### Activation markers

Splenocytes from CIA mice were stained with anti-CD3, anti-CD4, anti-CD8, anti-CD44 and anti-CD62L antibodies, and the surface markers for activation in CD4^+ ^and CD8^+ ^T cells were analyzed using an LSR II flow cytometer (BD).

### ELISA for serum anti-CII antibodies and their isotypes

Serum samples were collected from different groups of immunized mice at different days postimmunization (days 0, 14, 28, 35 and 42), the levels of anti-CII antibody in different isotypes were measured by ELISA as described in our previous studies [25]. Briefly, ELISA plates were coated with bovine CII (0.5 μg/well). After the plates were blocked with 5% fetal bovine serum-PBS, 1:800 diluted (with blocking buffer) mouse sera were added to duplicate wells and incubated for 2 hours at room temperature. The plates were then washed, and biotin-conjugated goat anti-mouse immunoglobulin G (IgG), IgG1 and IgG2a were added at a dilution of 1:2,000 for 1 hour, followed by the addition of streptavidin-horseradish peroxidase (Sigma-Aldrich). The plates were then developed, and antibody levels were measured as described previously [24].

### Quantitative real-time PCR

Total RNA was isolated from joints by using the RNeasy Mini Kit (QIAGEN, Valencia, CA, USA). Genomic DNA was removed by digestion with DNase I. cDNA was synthesized using the iScript cDNA Synthesis Kit (Bio-Rad Laboratories, Hercules, CA, USA), and gene expression was examined using the Stratagene Mx3005P QPCR System with Brilliant SYBR Green QPCR Master Mix reagent (both from Agilent Technologies, Inc, Santa Clara, CA, USA). The data were normalized to a *gapdh *reference [26]. The forward and reverse primers for *il17 *and *gapdh *were as follows [27]: *il1*7, TTTAACTCCCTTGGCGCAAAA, CTTTCCCTCCGCATTGACAC; and *gapdh*, TTCACCACCATGGAGAAGGC, GGCATGGACTGTGGTCATGA.

### Intracellular Foxp3 and cytokine staining

Single-cell suspensions were prepared from spleens, lymph nodes or thymi and stained with surface CD4 and CD25. After being fixed with freshly prepared fixation/permeabilization solution at 4°C for at least 30 minutes in the dark, cells were blocked with Mouse BD Fc Block (BD) in permeabilization buffer at 4°C for 15 minutes and stained with anti-Foxp3 antibody at 4°C for 30 minutes. For intracellular cytokine staining, cells were stimulated with PMA plus ionomycin in the presence of brefeldin A for 4 hours. Cytokine production was measured as described previously.

### Suppression assay

Enriched CD4^+ ^T cells were sorted into a CD4^+^CD25^+ ^subset as the regulatory cells and a CD4^+^CD25^- ^subset as the responder cells. T-cell-free splenocytes treated with mitomycin C were used as antigen-present cells (APCs). The responder cells were cultured with the regulatory cells at ratios of 1:1, 1:0.5, 1:0.25 and 1:0.125 or without the regulatory cells as the positive control in the presence of 2 μg/ml anti-CD3 antibody for 3 days in 96-well U-bottom plates, and APCs were added with the responder cells at a 5:1 ratio. After pulsing with 1 μCi of [^3^H]thymidine per well, proliferation was measured as radioactivity incorporation. The suppressive efficiency was calculated using the following formula: percentage suppression = (cpm of positive control) - (cpm of experiment)/(cpm of positive control) × 100.

### Adoptive transfer

CD4^+^RFP^+ ^Tregs were sorted from the spleens of B6 Thy1.1 IL-10 dominant-negative receptor Tg and Wt mice and were transferred into B6 Thy1.2 Wt mice at equal cell numbers. One day after transfer mice were immunized with CFA and bovine CII as described above. One week after immunization Foxp3 expression in transferred cells was analyzed by checking the levels of RFP.

### Histopathologic assessment

On day 58 after CIA induction, mice were killed and their paws were dissected free and used for histologic examination. The joints were immediately fixed in 10% buffered formalin and decalcified in a decalcifying solution for about 3 to 5 days [24]. The tissues were then processed and embedded in paraffin. Five-micrometer tissue sections were prepared and stained with H & E using standard methods. All sections were stained at the Yale Pathology Laboratory. Sections were blindly observed and scored by histologists. Five arthritis severity factors were assessed using a grading scale from 0 (normal) to 5 (severe), based on a previously described scoring system [28].

### Statistical analysis

Data presented are means ± SEM. Statistical testing was two-tailed with a 5% level of significance. A two-tailed nonparametric paired test (Wilcoxon signed-rank test) was used to analyze the arthritis and histologic scores. Student's paired or unpaired *t*-test was used for all other statistical analyses (InStat version 2.03 software; GraphPad Software, San Diego, CA, USA).

## Results

### Blocking IL-10 signaling in T cells rendered mice highly susceptible to collagen-induced arthritis

**Figure 1:**
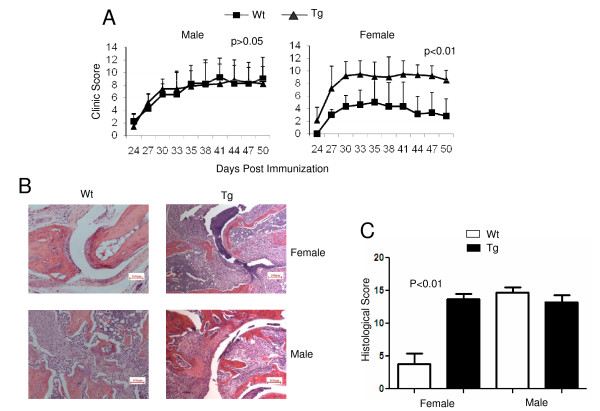
**IL-10 receptor dominant-negative transgenic mice were highly susceptible to collagen-induced arthritis (CIA)**. **(A) **Sex- and age-matched DBA/1J transgenic (Tg) and wild-type (Wt) littermate control mice were immunized with bovine type II collagen as described in "Materials and methods." The clinical arthritis score was observed, scored and recoded daily. Left (Wt males, *n *= 10, Tg males, *n *= 9; *P *> 0.05). Right (Wt females, *n *= 12, Tg females, *n *= 13; *P *< 0.01). The results of one of three repeated experiments are shown. **(B) **Representative H & E-stained sections of paw joints from Wt and Tg mice on day 50 postimmunization in CIA animals are shown (original magnification, ×10; scale bar, 100 μm). **(C) **Histological scores of paw joints on day 50 postimmunization. Each feature of arthritis (joint space exudate, synovitis, pannus formation and cartilage and bone degradation) was graded from 0 (normal) to 5 (severe). The values given are means and SEM (*n *= 4 per group; females, *P *< 0.01, and males, *P *> 0.05). The results are representative of two experiments.

To define the role of IL-10 signaling in T cells in the pathogenesis of CIA, we employed newly generated IL-10 receptor dominant-negative Tg mice in which T cells expressed high levels of Tg IL-10Ra that were truncated to remove its signaling domain [21]. Tg mice were backcrossed with DBA/1 mice 10 times to develop Tg mice on the CIA-susceptible background (DBA/1, H-2^q^). Four groups of DBA/1 mice (male Wt or Tg and female Wt or Tg) from the same cohort of littermates at age 8 weeks were immunized with bovine CII to induce arthritis using the standard CIA protocol described in our previous studies. It has been observed that even in the susceptible strain of mice, such as DBA/1 (H-2^q^), female mice are more resistant than male mice, with a lower incidence and less severity of CIA [28]. As expected, our experiment showed that male mice were highly susceptible to and females relatively resistant to CIA (Figure 1A). All the male Wt mice developed progressive disease (with 100% incidence) and had a mean arthritis score of 8.25 ± 2.88 (*n *= 10) on day 35 postimmunization. In contrast, all the female Wt mice developed visible arthritis (also 100% incidence) with a significantly lower mean arthritis score of 5.00 ± 4.10 (*n *= 12) on day 35 postimmunization. Interestingly, female Tg mice developed severe progressive disease and had a mean arthritis score of 8.78 ± 2.33 (*n *= 10), slightly higher than those parameters in male Wt mice and male Tg mice (7.89 ± 2.49, *n *= 12). Further evidence in support of increased arthritis severity in female Tg mice was obtained by histopathologic analysis. On day 50 postimmunization, female Tg mice exhibited robust pannus formation and significant articular cartilage erosion, with pannus eroding and replacing the articular cartilage overlying the bone. In contrast, Wt female mice exhibited well-preserved joint spaces and articular cartilage surfaces, with minimal pannus formation. Among male mice, both Wt and Tg exhibited extensive pannus formation and destruction of bone and cartilage. One representative example is shown in Figure 1B. At 50 days postimmunization, the histologic score of Wt female mice was 3.8 ± 1.4 of a possible score of 25. All the other groups showed similar scores (Figure 1C) (female Tg, 13.7 ± 0.6; male Wt, 14.7 ± 0.8; male Tg, 13.3 ± 1.0, respectively; *n *= 4 mice in each group). Because the major difference in CIA disease severity was observed between female Wt and Tg mice rather than male Wt and Tg mice, only female mice were used for the rest of our study. Thus our data suggest that IL-10 signaling in T cells plays a critical role in suppressing the pathogenesis of CIA.

### IL-10 signaling in T cells controls T-cell activation and proliferation responses in collagen-induced arthritis mice

**Figure 2:**
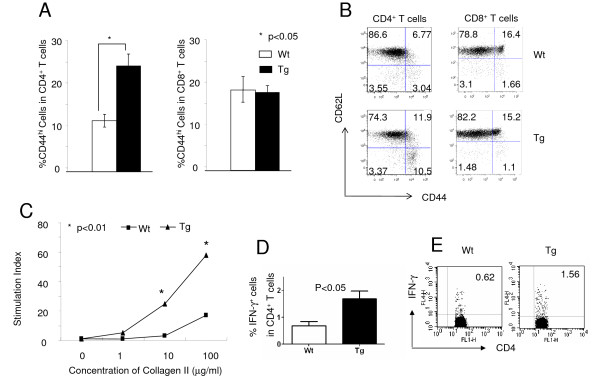
**Altered T-cell phenotype in IL-10 dominant-negative receptor transgenic mice upon induction of collagen-induced arthritis**. **(A) **Sex- and age-matched DBA/1 transgenic (Tg) and wild-type (Wt) littermate control mice were immunized with type II collagen as described in "Materials and methods." Cells from draining lymph nodes in female DBA/1 mice on day 28 postimmunization were analyzed for CD44 and CD62L expression after gating on CD4^+ ^(left) or CD8^+ ^T cells (right). Wt, *n *= 3, Tg, *n *= 3; *P < 0.05. **(B) **One representative stain of CD44, CD62L in CD3^+^CD4^+ ^(left) and CD3^+^CD8^+ ^(right) T cells of three repeated experiments is shown. **(C) **The same sources of cells described above were cultured with different concentrations of collagen for 3 days. After pulsing with 1 μCi of [^3^H]thymidine per well for the last 18 hours, proliferation was measured as radioactivity incorporation in counts per minute (cpm). Stimulation Index = cpm experiment/cpm control (concentration of collagen = 0). The results of one representative experiment are shown. **(D) **The cells were also stimulated with phorbol 12-myristate 13-acetate (50 ng/ml) and ionomycin (500 ng/ml) in the presence of BD GolgiPlug for 4 hours then stained with CD4 and intracellular IFN-γ. The statistical analysis for the percentage of IFN-γ^+ ^cells in the CD3^+^CD4^+ ^gate is shown (Wt, *n *= 3, Tg, *n *= 3; *P *< 0.05). One representative stain is shown **(E)**.

Activation and cytokine production of CD4^+ ^T cells play an important role in the pathogenesis of CIA. To define the underlying mechanisms of increased arthritis severity in female Tg mice, T-cell activation and proliferation in CIA mice were first analyzed. Cells from draining lymph nodes on day 50 postimmunization were stained with anti-CD3, anti-CD4, anti-CD8, anti-CD44 and anti-CD62L antibodies. After gating on CD3^+ ^T cells, the CD44^hi ^T cells were recognized as activated cells. Activated CD4^+ ^was found to be significantly higher in Tg mice than in Wt mice (Figure 2A, left), whereas activated CD8^+ ^in Tg and Wt mice appeared to be comparable (Figure 2A, right), implying that IL-10 signaling in T cells controlled CD4^+ ^T cell activation in inflammatory response. One representative dot plot is shown (Figure 2B). To test the impact of IL-10 signaling in antigen-specific T-cell proliferative response in immunized mice, cells recovered as described above were cultured in 96-well U-bottom plates in the presence of different concentrations of CII for 3 days, followed by pulsing with 1 μCi of [^3^H]thymidine per well for the last 18 hours, and proliferation was measured as radioactivity incorporated (in cpm). Cells from Tg mice showed significantly increased proliferative responses to specific antigen (Figure 2C), suggesting a critical role of IL-10 signaling in T cells in controlling lymphocyte cell proliferation in CIA. To further test whether IL-10 signaling in T cells would affect T-cell differentiation in CIA, we also analyzed IFN-γ-producing CD4^+ ^T cells in draining lymph nodes upon immunization and found elevated percentages of IFN-γ^+^CD4^+ ^cells in Tg mice (Figure 2D), suggesting that IL-10 signaling suppressed the pathogenic Th1 response. One representative dot plot is shown (Figure 2E). Interestingly, no significant differences were observed in terms of the numbers and the activation states of γδ T cells in the spleens and draining lymph nodes of Wt and Tg mice (data not shown).

### Impaired suppressive function of regulatory T cells from transgenic mice

**Figure 3:**
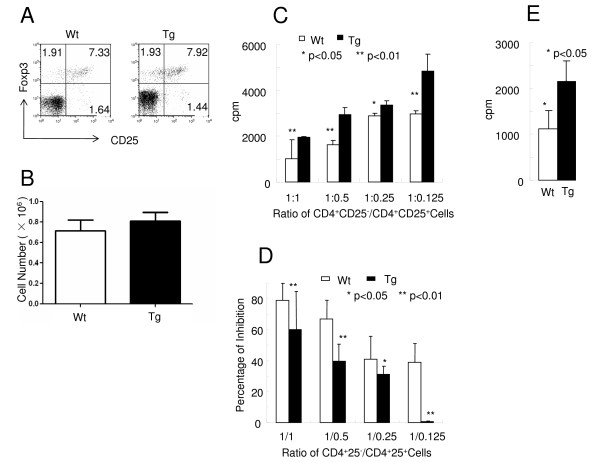
**Impaired suppressive function of regulatory T cells from transgenic mice**. **(A) **Splenocytes from C57BL/6 mice were stained with anti-CD4 and anti-CD25 antibodies, which was followed by Foxp3 intracellular staining, as described in "Materials and methods." The dot plots of CD25^+ ^and Foxp3^+ ^proportions after gating on CD3^+^CD4^+ ^T cells are shown. The results shown are from one of three representative experiments. **(B) **The absolute number of regulatory T cells (Tregs) (CD4^+^CD25^+^Foxp3^+ ^cells) from the spleens of C57BL/6 mice were calculated and are shown here (*n *= 3 mice for each group; *P *> 0.05). **(C) **CD4^+ ^T cells from the spleens of B6 wild-type (Wt) or transgenic (Tg) mice were enriched and sorted into a CD4^+^CD25^+ ^subset as the regulatory cells and a CD4^+^CD25^- ^subset from Wt C57BL/6 as the responder cells. The number of responder cells was fixed at 5 × 10^4^. The inhibition function of Tregs was measured by *in vitro *assay as described in "Materials and methods." The percentage inhibition by different populations of Tregs was calculated by using the formula described in "Materials and methods." cpm, counts per minute. **(D) **The results shown are from one of three representative experiments. **(E) **B6 Wt and Tg mice were crossed with Foxp3-RFP mice. CD4^+ ^T cells from the spleens of Wt or Tg red fluorescent protein (RFP) mice were enriched and sorted into a CD4^+^RFP^+ ^subset as the regulatory cells and a CD4^+^RFP^- ^subset from Wt C57BL/6 as the responder cells. The responder cells (5 × 10^4/^well) were cocultured with an equal number of regulatory cells for the inhibition assay described above. The results from one of three representative repeated experiments are shown.

It has been demonstrated that CD4^+^Foxp3^+ ^Tregs play important roles in the suppression of CIA in animal models and human studies [9,10]. To define the role of IL-10 signaling in T cells in the development of naturally occurring Tregs (nTregs), the percentage and absolute number of CD4^+^CD25^+^Foxp3^+ ^T cells in DBA/1 and B6 Tg and Wt mice were analyzed. Very similar percentages and numbers of CD25^+^Foxp3^+ ^T cells were found in both Wt and Tg mice (Figures 3A and 3B), indicating that IL-10 signaling in T cells was not required for the development of nTregs. Similar results were observed in DBA/1 mice upon immunization (data not shown). To further define the function of Tregs, a standard *in vitro *T-cell suppression assay was performed. CD4^+^CD25^+ ^T cells were sorted from B6 Wt or Tg mice as Tregs and CD4^+^CD25^- ^T cells from B6 Wt mice as responders. Tregs from both Wt and Tg mice were anergic to TCR activation (data not shown). Interestingly, the suppressor function of Tregs from Tg mice was significantly impaired in comparison to those from Wt mice (Figures 3C and 3D). To rule out the contamination of activated CD4^+ ^T cells in the Treg population when using CD25 as the marker of Tregs, RFP-Foxp3 reporter mice [23] were used to sort Foxp3^+ ^regulators and Foxp3^- ^responders and the suppress assay mentioned above was repeated. Similar results were obtained (Figure 3E). Our results demonstrate that IL-10 signaling in T cells is not required for Treg development, but rather for T cells' suppressive function.

### IL-10 signaling in T cells is required for maintenance of Foxp3 expression

**Figure 4:**
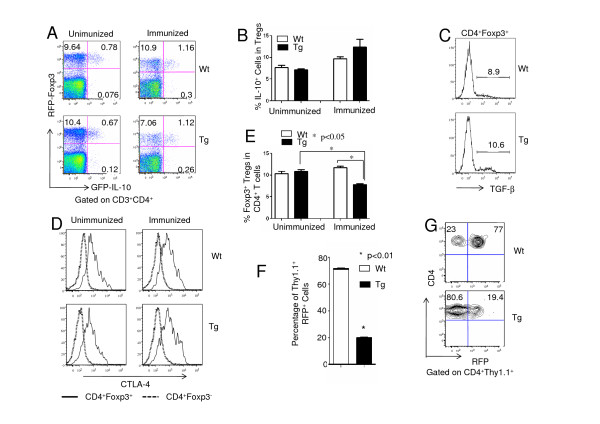
**IL-10 signaling is required for the maintenances of Foxp3 expression**. **(A) **IL-10 GFP and Foxp3 red fluorescent protein (RFP) dual-reporter mice (Tiger-Foxp3- internal ribosomal entry site-mRFP (Tiger-FIR) mice on C57BL/6 background) were crossed with B6 wild-type (Wt) or transgenic (Tg) mice, and these mice were then also immunized with type II collagen (CII) according to the same protocol that we used for collagen-induced arthritis. Upon gating on CD4^+ ^T cells, cells from the lymph nodes of unimmunized Wt and Tg mice were analyzed for RFP and GFP expression (left). Cells from draining lymph nodes of Wt and Tg mice on day 28 after immunization with CII and Complete Freund's Adjuvant (CFA) were also analyzed (right). The results of one representative experiment are shown. **(B) **The percentages of CD4^+^GFP^+ ^cells among CD4^+^RFP^+ ^cells in Wt and Tg mice described in (A) are shown (three mice in each group). Tregs, regulatory T cells. **(C) **Splenocytes from B6 Wt and Tg mice (five mice in each group) were stimulated with phorbol 12-myristate 13-acetate and ionomycin for 4 hours. The expression levels of Foxp3 and transforming growth factor β (TGF-β) were measured by intracellular staining. A representative example is shown for one of three repeated experiments. **(D) **CTLA4 was analyzed by intracellular staining in CD3^+^CD4^+^Foxp3^+ ^and CD3^+^CD4^+^Foxp3^- ^cells from the lymph nodes of unimmunized and immunized C57BL/6 Wt and Tg mice (day 28 after immunization with CII and CFA). The results shown are from one of three repeated experiments. **(E) **B6 Wt and Tg Foxp3 RFP mice used in (A) were immunized with CII, with CFA or left untreated, and the percentage of RFP^+ ^cells among gated CD4^+ ^cells in Wt and Tg mice are shown (three mice in each group). **(F) **RFP^+^CD4^+ ^T cells were sorted from B6 Thy1.1 Wt and Tg mice, transferred to B6 Thy1.2 Wt recipient mice and immunized with CII and CFA. One week later the level of Foxp3 expression in Thy1.1^+ ^cells from the host mice was analyzed on the basis of the percentage of RFP^+ ^cells (three mice in each group; *P *< 0.05). **(G) **The results of one representative experiment of the three repeated experiments described in (F) are shown.

To define the mechanisms underlying the reduced suppressive function of Tregs in Tg mice, we first analyzed IL-10 production in Tregs. For this purpose, we used IL-10-Foxp3 double-reporter mice [22,23]. The percentage of IL-10-expressing cells was similar between Wt and Tg mice in both unimmunized and immunized states (Figures 4A and 4B), indicating that IL-10 signaling in T cells was not required for IL-10 production by Tregs and that IL-10 was not related to the impaired suppressive function of Tg Tregs. We next analyzed the transforming growth factor (TGF)-β production by Tregs in Wt and the Tg mice. Lymphocytes from Wt and the Tg mice were stimulated with PMA and ionomycin for 4 hours in the presence of brefeldin A, and intracellular Foxp3 and TGF-β were stained in the CD4^+ ^T cell population. No significant differences in TGF-β production between Tregs from Wt and the Tg mice were detected (Figure 4C), suggesting that IL-10 signaling in T cells was not involved in TGF-β production. We then investigated whether IL-10 signaling in T cells would affect CTLA-4 expression level in Tregs. Cells from the lymph nodes of unimmunized and immunized Wt and the Tg mice were stained with Foxp3 and intracellular CTLA-4. CTLA-4 was highly expressed in CD4^+^Foxp3^+ ^T cells, but not in CD4^+^Foxp3^- ^T cells; however, no significant differences were found in the levels of CTLA-4 in Tregs from Wt and Tg mice (Figure 4D). Because Foxp3 has been reported to be the master transcription factor controlling Treg development and determining the suppressive function of Tregs [29-31], we studied whether IL-10 signaling played any role in maintaining the stable expression of Foxp3. Similarly to the results shown in Figure 3A, the percentage of Foxp3^+ ^T cells was very similar between Wt and Tg mice (Figure 4A, left). Interestingly, it decreased by 30% upon immunization (Figure 4A, right, and Figure 4E). A similar phenomenon was observed in DBA/1 mice using intracellular staining (data not shown). To define whether IL-10 signaling was critical for the maintenance of Foxp3 expression in Tregs, CD4^+^RFP^+ ^T cells from Wt and Tg Thy1.1 mice were sorted and transferred to Thy1.2 Wt mice, followed by immunization with CII and CFA. One week later the transferred cells were analyzed for expression of RFP. Transferred Foxp3^+ ^cells were observed to become RFP^- ^cells in both Tg and Wt Tregs (Figures 4F and 4G). However, Tregs from Tg mice showed significantly higher percentages of RFP^- ^cells than those from Wt mice, indicating that IL-10 signaling in Tregs was critical to maintaining the level of Foxp3 expression upon immunization.

### Increased IL-17^+ ^γδ T cells and joint IL-17 expression in transgenic mice

**Figure 5:**
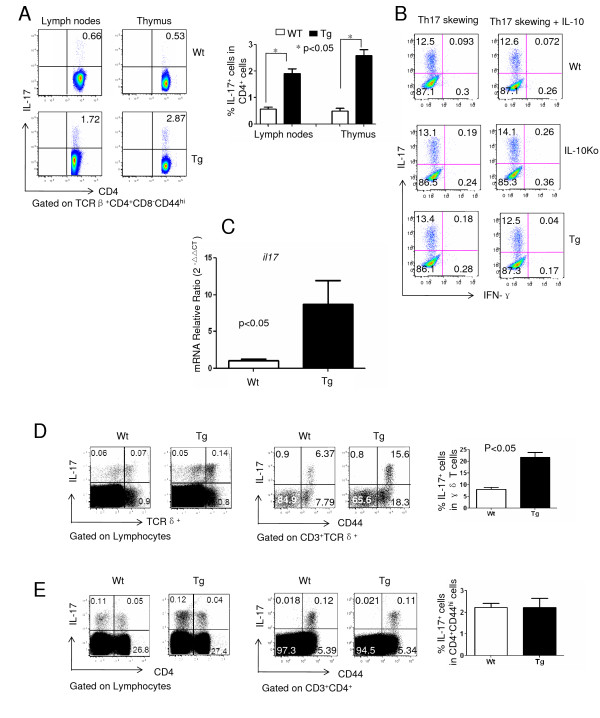
**Increased IL-17 mRNA expression in the joints of transgenic mice**. **(A) **Cells from the lymph nodes and thymi of untreated B6 wild-type (Wt) or transgenic (Tg) mice were stimulated with phorbol 12-myristate 13-acetate (50 ng/ml) and ionomycin (500 ng/ml) in the presence of BD GolgiPlug for 4 hours, followed by staining with TCRβ, CD4, CD8, CD44 and intracellular IL-17. One representative stain is shown (right). The statistical analysis of the percentage of IL-17^+ ^cells in TCRβ^+^CD4^+^CD8^-^CD44^hi ^cells (three mice in each group) is shown (left). **(B) **Naïve CD4^+ ^T cells (CD4^+^CD44^low^CD62L^hi^) from B6 Wt, IL-10-knockout (IL-10Ko) and Tg mice were sorted and stimulated with coated anti-CD3 and anti-CD28 antibodies under Th17 differentiation conditions in the presence or absence of IL-10 (100 μg/ml). Seven days later IL-17 production was measured by intracellular staining. The results of one of three representative experiments are shown. **(C) **The relative levels of IL-17 mRNA from the joints of DBA/1J Wt and Tg mice with collagen-induced arthritis (on day 50 postimmunization) were measured by real-time PCR. **(D) **Cells from draining lymph nodes of immunized C57BL/6 Wt or Tg mice (on day 7 postimmunization) were stimulated with the same procedure described above for IL-17 intracellular staining. One representative stain showing lymphocytes (left) or CD3^+^TCRδ^+ ^T cells (middle) upon gating is shown. The statistical analysis for the percentage of IL-17^+ ^cells in CD3^+^TCRδ^+ ^cells (three mice in each group) is shown (right). **(E) **The same population of cells described in (D) was also stimulated for IL-17 intracellular staining upon gating on lymphocytes (left) or CD4^+ ^T cells (middle). Statistics analysis for the percentage of IL-17^+ ^cells in TCRβ^+^CD4^+^CD8^-^CD44^hi ^cells (three mice in each group) is shown (right).

IL-17 is a critical cytokine in the pathogenesis of CIA [32]. To define the role of IL-10 signaling in T cells on IL-17 production by CD4^+ ^T cells, we first compared the IL-17^+^CD4^+ ^T cells from unimmunized mice. We found that Tg mice had increased Th17 cells in both peripheral and central lymph organs (Figure 5A), suggesting that IL-10 signaling negatively regulates naturally occurring Th17 cell development in steady state. To further define the role of IL-10 in Th17 cell differentiation, we sorted naïve CD4^+ ^T cells (CD4^+^CD44^lo ^CD62L^hi ^cells) from Tg as well as IL-10-knockout (IL-10Ko) mice versus Wt controls cultured under Th17 differentiation conditions in the presence or absence of IL-10, and the cells were then used to analyze cytokine production. No significant difference was found in terms of the percentage of IL-17-producing cells among Tg, Ko and Wt mice with or without IL-10, indicating that IL-10 signaling had no effect on inducible Th17 cell differentiation (Figure 5B). To define the role of IL-17 in local inflammatory response, the level of IL-17 mRNA (*il17*) in the joints of CIA mice was analyzed using real-time PCR. We found that joint tissues from Tg mice expressed significantly higher levels of *il17 *than Wt mice did (Figure 5C), which was consistent with the severity of arthritis. To define the potential sources of IL-17 in the joints, cells from draining lymph nodes of CIA mice (Wt and Tg mice) were analyzed for IL-17A production. Interestingly, significantly higher percentages of γδ T cells from Tg mice produced IL-17 than those from Wt mice (Figure 5D), especially CD44^hi ^γδ T cells. In contrast, we did not find significant differences in the number of IL-17-producing CD4^+ ^T cells (Figure 5E), suggesting γδ T cells as the primary source of IL-17 in the inflammatory joints.

### IL-10 signaling in T cells has no effect on antigen-specific antibody response

**Figure 6:**
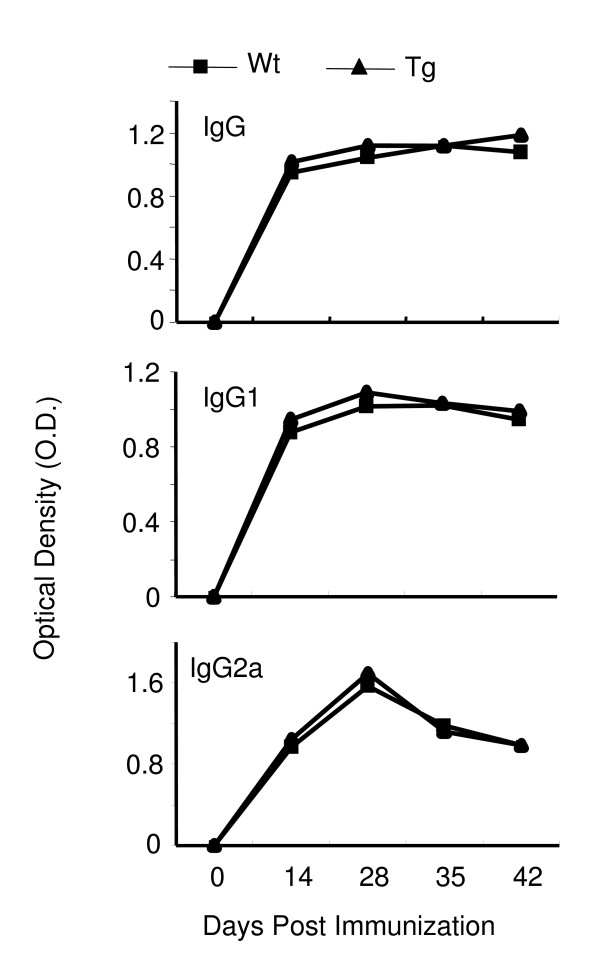
**IL-10 signaling in T cells has no effect on type II collagen-specific antibody production**. Serum samples from DBA/1J Wt or Tg mice (six mice in each group) were collected on days 0, 14, 21, 35 and 42 after collagen-induced arthritis induction, and collagen-specific antibodies (immunoglobulin G (IgG), IgG1 and IgG2a) were detected by ELISA. The results of one representative experiment are shown.

Antibody production against CII is an important factor in the development of CIA [33]. To define the impact of IL-10 signaling in T cells on collagen-specific antibody response, serum samples were collected from naïve mice as well as from different groups of immunized mice at different time points after immunization (days 0, 14, 28, 35 and 42), and the levels of anticollagen antibodies were analyzed by ELISA as described in our previous studies [24]. The level of collagen-specific antibody in serum increased upon immunization and was maintained at high levels between days 14 and 42. Surprisingly, there was no difference in collagen-specific antibodies, including the total IgG, IgG1 and IgG2a isotypes, among Wt and Tg mice (Figure 6). These results suggest that blocking IL-10 signaling in T cells has no direct effect on collagen-specific antibody production by B cells in a CIA model.

## Discussion

The CIA has been used as a model for studying the pathogenesis and potential application of novel therapeutic agents for human RA. Both T and B cells are essential for the development of RA [34,35], and IL-10 plays different roles in regulating the function of T and B cells [36]. It is therefore important to study the unique role of IL-10 signaling in T cells (without affecting B cells) in the pathogenesis of CIA. In this study, we demonstrated that IL-10 signaling in T cells play a critical role in the pathogenesis of CIA by affecting the function of Tregs by stabilizing the expression of Foxp3.

To target IL-10 signaling selectively in T cells, a unique IL-10 receptor dominant-negative Tg mouse was recently developed with the Tg under the control of CD4 promoter, which lacks the CD8 silencer element and therefore is expressed in both CD4^+ ^and CD8^+ ^T cells [21]. In these mice, CD4^+ ^and CD8^+ ^T cells expressed high levels of the Tg IL-10Ra, which lacks the cytoplasmic domain. As this receptor functions as a dominant-negative receptor, its responsiveness to IL-10 is attenuated in these T cells. Using these mice, we demonstrated that Tg mice developed more severe arthritis upon induction in the absence of IL-10 signaling (Figure 1). This was especially true for female mice. Even in susceptible strains, female Wt mice are more resistant than males, and the underlying mechanisms have not been studied systematically. Our observation that blockade of IL-10 signaling in T cells rendered female mice highly susceptible to CIA may shed some light on the molecular mechanisms of the gender differences. Of course, we did not rule out the possibility that IL-10 signaling might also affect the male mice if these mice were immunized with lower doses of CII and/or subdoses of adjuvant. Further studies are needed to explore this possibility.

CD4^+ ^T cells play an essential role in the pathogenesis of CIA as well as RA. Autoreactive T cells are normally controlled by Tregs and other regulatory mechanisms under normal circumstances, whereas in autoimmune diseases Treg dysfunction causes autoreactive T-cell proliferative responses and tissue damage [37]. As one of the key suppressive cytokines, IL-10 plays an important role in controlling T-cell responses. Indeed, though T cells were unable to respond to IL-10, they were more activated and proliferated more vigorously upon CIA induction, with a a more pronounced Th1 response (Figure 2). Our data suggest that a potential mechanism for this enhanced T-cell response could be the dysfunction of Tregs. Although IL-10 signaling in T cells was not required for the development of nTregs (Figures 3A and 4A), we clearly have demonstrated that the suppressive function of Tregs was significantly impaired in the absence of IL-10 signaling (Figures 3C and 3D).

Several elements are critical for the function of Tregs, including IL-10, TGF-β and CTLA-4 [38]. To define which factor was critical for the function of Tregs in connection of IL-10 signaling in T cells, we analyzed the levels of IL-10, TGF-β and CTLA-4 in Tregs of Wt and the Tg mice. To our surprise, no significant differences were detected between these two populations of Tregs (Figure 4), implying that IL-10 signaling in T cells was not required for the expression of these factors.

Foxp3 is a critical factor that determines not only the development and differentiation but also the suppression function of Tregs. Expression of Foxp3 can be regulated by multiple factors, including inflammatory cytokines [39]. One of the striking findings of our study is that we have demonstrated that in the absence of IL-10 signaling in T cells, the expression levels of Foxp3 were reduced upon immunization, on the basis of both *ex vivo *studies (Figure 4A) and Foxp3-RFP T-cell adoptive transfer experiments (Figures 4F and 4G). This phenotype was observed in C57BL/6 mice as well as in DBA/1 mice, suggesting that Foxp3 downregulation in Tg mice upon immunization may be associated with the disease mechanism in CIA (data not shown). By taking advantage of our Foxp3-RFP reporter mice, we were able to isolate a pure population of Tregs and track these cells in the congenic hosts. We speculated that upon immunization, inflammatory cytokines might target Tregs and regulate expression of Foxp3. One of the critical functions of IL-10 signaling in these Tregs probably was to maintain the expression of this key molecule. Therefore, our results collectively indicate that IL-10 signaling in T cells is critical for the function of Tregs by maintaining the expression level of Foxp3. Although *Foxp3 *is an X-linked gene, no significant difference was observed in the expression levels of Foxp3 between male and female mice (data not shown). Further studies are needed to define the molecular mechanisms underlying how the IL-10 signaling pathway maintains the expression of Foxp3.

IL-17 has been defined as a critical cytokine that mediates autoimmune inflammation, especially in CIA and RA [40]. This has been evidenced by a series of studies using different gene-deficient mice, including p19^-/-^, inducible costimulatory molecule (ICOS^-/-^) and IL-17^-/- ^mice [18,19,40]. Indeed, the expression levels of IL-17A were found to be significantly increased in the joints of Tg mice, which correlated with severe arthritis (Figure 5). Interestingly, the increased levels of IL-17 were not derived from CD4^+ ^T cells; rather, they were most likely derived from γδ T cells (Figures 5D and 5E). Our results are consistent with those of previous studies in that Vγ4 γδ T cells provided IL-17A in the pathogenesis of CIA [41]. How does IL-10 signaling in CD4^+ ^T cells affect the production or recruitment of IL-17A-producing γδ T cells is unclear at present. Several possibilities might explain our findings. IL-10 signaling promoted both thymic and peripheral CD4^+ ^T cells to produce IL-17A in naïve, but not in immunized, mice (Figure 5A), indicating a possibility that IL-10 signaling might change the threshold of IL-17 expression by CD4^+ ^T cells. The increased percentage of IL-17^+ ^γδ T cells in the draining lymph nodes of CIA mice could be a result of more local differentiation, more recruitment from other tissues, or both. Whether increased IL-17 production from CD4^+ ^T cells in naïve mice has any impact on IL-17 production by γδ T cells upon immunization remains to be determined.

We have to emphasize that IL-10 signaling in T cells did not affect T-cell-B-cell collaboration or the production of CII-specific antibodies (Figure 6). IL-10 has been defined as a B-cell-stimulating factor and can promote antibody production [42,43]. Because the function of T cells but not B cells was affected in Tg mice [21], it was not surprising that we saw no differences in autoantibody production between Wt and Tg mice. More generally, this Tg mouse may provide a unique model in which to study the dysfunction of T cells without affecting antibody responses.

Our study clearly demonstrates that IL-10 signaling in T cells is critical for the pathogenesis of CIA by maintaining the level of Foxp3 and consequently contributing to the dysfunction of Tregs as well as to the increased expression level of the innate source of IL-17A by γδ T cells. Enhancement of IL-10 signaling in T cells may regulate the function of Tregs, which can dampen the harmful autoimmune responses.

## Conclusions

Our results demonstrate a critical role of IL-10 signaling in T cells in the pathogenesis of CIA. Without IL-10 signaling, T cells, especially Tregs, lost their suppressive function, which in turn failed to control CD4^+ ^T-cell activation. Enhancement of IL-10 signaling in T cells might serve as a therapeutically sound approach to the treatment of RA as well as other autoimmune inflammatory diseases.

## Abbreviations

CFA: Complete Freund's Adjuvant; CIA: collagen-induced arthritis; ELISA: enzyme-linked immunosorbent assay; GFP: green fluorescent protein; H & E: hematoxylin and eosin; IFA: Incomplete Freund's Adjuvant; IFN-γ: interferon-γ; IL: interleukin; PCR: polymerase chain reaction; TCR: T-cell receptor; Tg: transgenic; Wt: wild type.

## Competing interests

The authors declare that they have no competing interests.

## Authors' contributions

JT performed most of the animal experiments as well as the *in vitro *experiments. MK prepared the IL-10 receptor dominant-negative transgenic mice. JH performed T-cell proliferation assays and intracellular cytokine staining. JC, one of the senior authors, engaged in discussion and made suggestions regarding the experiments. RF, one of the senior authors, was responsible for the design of the experiments and discussed the data. ZW helped to perform the animal experiments. ZHong helped to perform *in vitro *assays. LZ helped with the data analysis and interpretation of the results. ZY, the corresponding author, designed the experiments and wrote the paper. ZHao performed the histological scoring. XJ assessed the H & E staining and took the histologic pictures. All authors read and approved the final manuscript.

## Authors' information

JT, PhD, associate research scientist; MK, PhD, postdoctoral fellow; JH, PhD candidate; JC, professor; RF, professor; ZW, assistant professor; ZH, professor; LZ, professor; ZY, professor; ZH, research associate; XJ, research associate.

## References

[B1] Feldmann M Brennan FM Maini RN Rheumatoid arthritis Cell 1996 85 307 310 10.1016/S0092-8674(00)81109-5 8616886

[B2] Courtenay JS Dallman MJ Dayan AD Martin A Mosedale B Immunisation against heterologous type II collagen induces arthritis in mice Nature 1980 283 666 668 10.1038/283666a0 6153460

[B3] McInnes IB Schett G Cytokines in the pathogenesis of rheumatoid arthritis Nat Rev Immunol 2007 7 429 442 10.1038/nri2094 17525752

[B4] Moore KW de Waal Malefyt R Coffman RL O'Garra A Interleukin-10 and the interleukin-10 receptor Annu Rev Immunol 2001 19 683 765 10.1146/annurev.immunol.19.1.683 11244051

[B5] Johansson AC Hansson AS Nandakumar KS Backlund J Holmdahl R IL-10-deficient B10.Q mice develop more severe collagen-induced arthritis, but are protected from arthritis induced with anti-type II collagen antibodies J Immunol 2001 167 3505 3512 11544344 10.4049/jimmunol.167.6.3505

[B6] Fiorentino DF Zlotnik A Mosmann TR Howard M O'Garra A IL-10 inhibits cytokine production by activated macrophages J Immunol 1991 147 3815 3822 1940369

[B7] Santin AD Hermonat PL Ravaggi A Bellone S Pecorelli S Roman JJ Parham GP Cannon MJ Interleukin-10 increases Th1 cytokine production and cytotoxic potential in human papillomavirus-specific CD8(+) cytotoxic T lymphocytes J Virol 2000 74 4729 4737 10.1128/JVI.74.10.4729-4737.2000 10775611 PMC111995

[B8] Rowbottom AW Lepper MA Garland RJ Cox CV Corley EG Interleukin-10-induced CD8 cell proliferation Immunology 1999 98 80 89 10.1046/j.1365-2567.1999.00828.x 10469237 PMC2326898

[B9] van Amelsfort JM Jacobs KM Bijlsma JW Lafeber FP Taams LS CD4^+^CD25^+ ^regulatory T cells in rheumatoid arthritis: differences in the presence, phenotype, and function between peripheral blood and synovial fluid Arthritis Rheum 2004 50 2775 2785 10.1002/art.20499 15457445

[B10] Kelchtermans H De Klerck B Mitera T Van Balen M Bullens D Billiau A Leclercq G Matthys P Defective CD4+CD25+ regulatory T cell functioning in collagen-induced arthritis: an important factor in pathogenesis, counter-regulated by endogenous IFN-gamma Arthritis Res Ther 2005 7 R402 415 10.1186/ar1500 15743488 PMC1065335

[B11] Morgan ME Sutmuller RP Witteveen HJ van Duivenvoorde LM Zanelli E Melief CJ Snijders A Offringa R de Vries RR Toes RE CD25+ cell depletion hastens the onset of severe disease in collagen-induced arthritis Arthritis Rheum 2003 48 1452 1460 10.1002/art.11063 12746920

[B12] Frey O Petrow PK Gajda M Siegmund K Huehn J Scheffold A Hamann A Radbruch A Brauer R The role of regulatory T cells in antigen-induced arthritis: aggravation of arthritis after depletion and amelioration after transfer of CD4+CD25+ T cells Arthritis Res Ther 2005 7 R291 301 10.1186/ar1484 15743476 PMC1065322

[B13] Groux H O'Garra A Bigler M Rouleau M Antonenko S de Vries JE Roncarolo MG A CD4+ T-cell subset inhibits antigen-specific T-cell responses and prevents colitis Nature 1997 389 737 742 10.1038/39614 9338786

[B14] Apetoh L Quintana FJ Pot C Joller N Xiao S Kumar D Burns EJ Sherr DH Weiner HL Kuchroo VK The aryl hydrocarbon receptor interacts with c-Maf to promote the differentiation of type 1 regulatory T cells induced by IL-27 Nat Immunol 2010 11 854 861 10.1038/ni.1912 20676095 PMC2940320

[B15] Harrington LE Hatton RD Mangan PR Turner H Murphy TL Murphy KM Weaver CT Interleukin 17-producing CD4+ effector T cells develop via a lineage distinct from the T helper type 1 and 2 lineages Nat Immunol 2005 6 1123 1132 10.1038/ni1254 16200070

[B16] Park H Li Z Yang XO Chang SH Nurieva R Wang YH Wang Y Hood L Zhu Z Tian Q Dong C A distinct lineage of CD4 T cells regulates tissue inflammation by producing interleukin 17 Nat Immunol 2005 6 1133 1141 10.1038/ni1261 16200068 PMC1618871

[B17] Hirota K Hashimoto M Yoshitomi H Tanaka S Nomura T Yamaguchi T Iwakura Y Sakaguchi N Sakaguchi S T cell self-reactivity forms a cytokine milieu for spontaneous development of IL-17+ Th cells that cause autoimmune arthritis J Exp Med 2007 204 41 47 10.1084/jem.20062259 17227914 PMC2118414

[B18] Nakae S Nambu A Sudo K Iwakura Y Suppression of immune induction of collagen-induced arthritis in IL-17-deficient mice J Immunol 2003 171 6173 6177 14634133 10.4049/jimmunol.171.11.6173

[B19] Murphy CA Langrish CL Chen Y Blumenschein W McClanahan T Kastelein RA Sedgwick JD Cua DJ Divergent pro- and antiinflammatory roles for IL-23 and IL-12 in joint autoimmune inflammation J Exp Med 2003 198 1951 1957 10.1084/jem.20030896 14662908 PMC2194162

[B20] Gu Y Yang J Ouyang X Liu W Li H Bromberg J Chen SH Mayer L Unkeless JC Xiong H Interleukin 10 suppresses Th17 cytokines secreted by macrophages and T cells Eur J Immunol 2008 38 1807 1813 10.1002/eji.200838331 18506885 PMC2733944

[B21] Kamanaka M Huber S Zenewicz LA Gagliani N Rathinam C O'Connor W Jr Wan YY Nakae S Iwakura Y Hao L Flavell RA Memory/effector (CD45RBlo) CD4 T cells are controlled directly by IL-10 and cause IL-22-dependent intestinal pathology J Exp Med 2011 208 1027 1040 10.1084/jem.20102149 21518800 PMC3092344

[B22] Kamanaka M Kim ST Wan YY Sutterwala FS Lara-Tejero M Galan JE Harhaj E Flavell RA Expression of interleukin-10 in intestinal lymphocytes detected by an interleukin-10 reporter knockin tiger mouse Immunity 2006 25 941 952 10.1016/j.immuni.2006.09.013 17137799

[B23] Wan YY Flavell RA Identifying Foxp3-expressing suppressor T cells with a bicistronic reporter Proc Natl Acad Sci USA 2005 102 5126 5131 10.1073/pnas.0501701102 15795373 PMC556008

[B24] Pan M Kang I Craft J Yin Z Resistance to development of collagen-induced arthritis in C57BL/6 mice is due to a defect in secondary, but not in primary, immune response J Clin Immunol 2004 24 481 491 15359107 10.1023/B:JOCI.0000040919.16739.44

[B25] You X Pan M Gao W Shiah HS Tao J Zhang D Koumpouras F Wang S Zhao H Madri JA Baker D Cheng YC Yin Z Effects of a novel tylophorine analog on collagen-induced arthritis through inhibition of the innate immune response Arthritis Rheum 2006 54 877 886 10.1002/art.21640 16508970

[B26] Livak KJ Schmittgen TD Analysis of relative gene expression data using real-time quantitative PCR and the 2(-Delta Delta C(T)) Method Methods 2001 25 402 408 10.1006/meth.2001.1262 11846609

[B27] Ishigame H Kakuta S Nagai T Kadoki M Nambu A Komiyama Y Fujikado N Tanahashi Y Akitsu A Kotaki H Sudo K Nakae S Sasakawa C Iwakura Y Differential roles of interleukin-17A and -17F in host defense against mucoepithelial bacterial infection and allergic responses Immunity 2009 30 108 119 10.1016/j.immuni.2008.11.009 19144317

[B28] Staite ND Richard KA Aspar DG Franz KA Galinet LA Dunn CJ Induction of an acute erosive monarticular arthritis in mice by interleukin-1 and methylated bovine serum albumin Arthritis Rheum 1990 33 253 260 10.1002/art.1780330215 2306293

[B29] Gavin MA Rasmussen JP Fontenot JD Vasta V Manganiello VC Beavo JA Rudensky AY Foxp3-dependent programme of regulatory T-cell differentiation Nature 2007 445 771 775 10.1038/nature05543 17220874

[B30] Fontenot JD Rasmussen JP Williams LM Dooley JL Farr AG Rudensky AY Regulatory T cell lineage specification by the forkhead transcription factor foxp3 Immunity 2005 22 329 341 10.1016/j.immuni.2005.01.016 15780990

[B31] Fontenot JD Gavin MA Rudensky AY Foxp3 programs the development and function of CD4+CD25+ regulatory T cells Nat Immunol 2003 4 330 336 12612578 10.1038/ni904

[B32] Korn T Bettelli E Oukka M Kuchroo VK IL-17 and Th17 Cells Annu Rev Immunol 2009 27 485 517 10.1146/annurev.immunol.021908.132710 19132915

[B33] Yanaba K Hamaguchi Y Venturi GM Steeber DA St Clair EW Tedder TF B cell depletion delays collagen-induced arthritis in mice: arthritis induction requires synergy between humoral and cell-mediated immunity J Immunol 2007 179 1369 1380 17617630 10.4049/jimmunol.179.2.1369

[B34] Peterman GM Spencer C Sperling AI Bluestone JA Role of gamma delta T cells in murine collagen-induced arthritis J Immunol 1993 151 6546 6558 8245484

[B35] Corthay A Johansson A Vestberg M Holmdahl R Collagen-induced arthritis development requires alpha beta T cells but not gamma delta T cells: studies with T cell-deficient (TCR mutant) mice Int Immunol 1999 11 1065 1073 10.1093/intimm/11.7.1065 10383939

[B36] Katsikis PD Chu CQ Brennan FM Maini RN Feldmann M Immunoregulatory role of interleukin 10 in rheumatoid arthritis J Exp Med 1994 179 1517 1527 10.1084/jem.179.5.1517 8163935 PMC2191503

[B37] Sakaguchi S Naturally arising CD4+ regulatory t cells for immunologic self-tolerance and negative control of immune responses Annu Rev Immunol 2004 22 531 562 10.1146/annurev.immunol.21.120601.141122 15032588

[B38] Sakaguchi S Ono M Setoguchi R Yagi H Hori S Fehervari Z Shimizu J Takahashi T Nomura T Foxp3+ CD25+ CD4+ natural regulatory T cells in dominant self-tolerance and autoimmune disease Immunol Rev 2006 212 8 27 10.1111/j.0105-2896.2006.00427.x 16903903

[B39] Murai M Turovskaya O Kim G Madan R Karp CL Cheroutre H Kronenberg M Interleukin 10 acts on regulatory T cells to maintain expression of the transcription factor Foxp3 and suppressive function in mice with colitis Nat Immunol 2009 10 1178 1184 10.1038/ni.1791 19783988 PMC2898179

[B40] Nurieva RI Treuting P Duong J Flavell RA Dong C Inducible costimulator is essential for collagen-induced arthritis J Clin Invest 2003 111 701 706 10.1172/JCI17321 12618524 PMC151904

[B41] Roark CL French JD Taylor MA Bendele AM Born WK O'Brien RL Exacerbation of collagen-induced arthritis by oligoclonal, IL-17-producing gamma delta T cells J Immunol 2007 179 5576 5583 17911645 10.4049/jimmunol.179.8.5576 PMC2768546

[B42] Go NF Castle BE Barrett R Kastelein R Dang W Mosmann TR Moore KW Howard M Interleukin 10, a novel B cell stimulatory factor: unresponsiveness of X chromosome-linked immunodeficiency B cells J Exp Med 1990 172 1625 1631 10.1084/jem.172.6.1625 2124252 PMC2188770

[B43] Stein SH Hart TE Hoffman WH Hendrix CL Gustke CJ Watson SC Interleukin-10 promotes anti-collagen antibody production in type I diabetic peripheral B lymphocytes J Periodontal Res 1997 32 189 195 10.1111/j.1600-0765.1997.tb01404.x 9085233

